# Artificial intelligence-assisted diagnosis and prognostication in low ejection fraction using electrocardiograms in inpatient department: a pragmatic randomized controlled trial

**DOI:** 10.1186/s12916-025-04190-z

**Published:** 2025-06-09

**Authors:** Dung-Jang Tsai, Chin Lin, Wei-Ting Liu, Chiao-Chin Lee, Chiao-Hsiang Chang, Wen-Yu Lin, Yu-Lan Liu, Da-Wei Chang, Ping-Hsuan Hsieh, Chien-Sung Tsai, Yuan-Hao Chen, Yi-Jen Hung, Chin-Sheng Lin

**Affiliations:** 1 Medical Technology Education Center, School of Medicine, National Defense Medical Center, Taipei, Taiwan Republic of China; 2 Department of Artificial Intelligence and Internet of Things, Tri-Service General Hospital, National Defense Medical Center, Taipei, Taiwan Republic of China; 3 Division of Cardiology, Department of Internal Medicine, Tri-Service General Hospital, National Defense Medical Center, Taipei, Taiwan Republic of China; 4 School of Pharmacy, National Defense Medical Center, Taipei, Taiwan Republic of China; 5 Division of Cardiovascular Surgery, Department of Surgery, Tri-Service General Hospital, National Defense Medical Center, Taipei, Taiwan Republic of China; 6 Department of Neurological Surgery, Tri-Service General Hospital, National Defense Medical Center, Taipei, Taiwan Republic of China; 7 Division of Endocrinology and Metabolism, Department of Internal Medicine, Tri-Service General Hospital, National Defense Medical Center, Taipei, Taiwan Republic of China

**Keywords:** Artificial intelligence, Electrocardiogram, Rapid response systems, Low Ejection Fraction, Randomized clinical trial, High-intensity care, Deep learning, Track and trigger system, Hospital information system, Electronic health records

## Abstract

**Background:**

Early diagnosis of low ejection fraction (EF) remains challenging despite being a treatable condition. This study aimed to evaluate the effectiveness of an electrocardiogram (ECG)-based artificial intelligence (AI)-assisted clinical decision support tool in improving the early diagnosis of low EF among inpatient patients under non-cardiologist care.

**Methods:**

We conducted a pragmatic randomized controlled trial at an academic medical center in Taiwan. 13,631 inpatient patients were randomized to either the intervention group (*n* = 6,840) receiving AI-generated ECG results or the control group (*n* = 6,791) following standard care. The primary outcome was the incidence of newly diagnosed low EF (≤ 50%) within 30 days following the ECG. Secondary outcomes included echocardiogram utilization rates, positive predictive value for low EF detection, and cardiology consultation rates. Statistical analysis included hazard ratios (HR) with 95% confidence intervals (CI) for time-to-event outcomes and chi-square tests for categorical variables.

**Results:**

The intervention significantly increased the detection of newly diagnosed low EF in the overall cohort (1.5% vs. 1.1%, HR 1.50, 95% CI: 1.11–2.03, *P* = 0.023), with a more pronounced effect among AI-identified high-risk patients (13.0% vs. 8.9%, HR 1.55, 95% CI: 1.08–2.21). While overall echocardiogram utilization remained similar between groups (17.1% vs. 17.3%, HR 1.00, 95% CI: 0.92–1.09), the intervention group demonstrated higher positive predictive value for identifying low EF among patients receiving echocardiogram (34.2% vs. 20.2%, *p* < 0.001). Post-hoc analysis revealed increased cardiology consultation rates among high-risk patients in the intervention group (29.3% vs. 23.5%, *p* = 0.027).

**Conclusions:**

Implementation of an AI-ECG algorithm enhanced the early diagnosis of low EF in the inpatient setting, primarily by improving diagnostic efficiency rather than increasing overall healthcare utilization. The tool was particularly effective in identifying high-risk patients who benefited from increased specialist consultation and more targeted diagnostic testing.

**Trial registration:**

ClinicalTrials.gov Identifier: NCT05117970.

**Supplementary Information:**

The online version contains supplementary material available at 10.1186/s12916-025-04190-z.

## Contributions to the literature

- This large randomized trial demonstrates the effectiveness of AI-ECG algorithms in improving early detection of low ejection fraction in routine hospital care
- The study shows AI can enhance diagnostic efficiency without increasing healthcare utilization
- Results provide a practical model for implementing AI tools in non-specialist settings

## Background

Heart failure affects over 6 million Americans, causing 1 million hospitalizations yearly [1]. This disease imposes significant health and economic burdens [2]. While current guidelines focus on symptom management, prevention and early intervention in left ventricular dysfunction is still important [3]. Asymptomatic left ventricular systolic dysfunction (ALVSD) is characterized by reduced LV systolic function without clinical heart failure symptoms. Early treatment initiation in patients with presumed ALVSD has been associated with improved outcomes [4–6].

While routine echocardiogram screening isn't recommended due to cost concerns [7], developing affordable, non-invasive tools to identify at-risk patients could be valuable. Previous research has underscored the utility of natriuretic peptides, a history of hypertension, myocardial infarction, and ECG findings as valuable biomarkers for the detection of reduced left ventricular ejection fraction (LVEF) within a community-based cohort [8, 9]. While the readily available and cost-effective ECG holds promise as an ideal tool for identifying patients warranting echocardiogram examinations, the critical issue of limited ECG interpretation skills among general practitioners [10] must be addressed. Consequently, the development of an artificial intelligence (AI) algorithm capable of enhancing general practitioners'ECG reading proficiency could offer an optimal screening solution within general medical practice and non-cardiology departments [11]. We previously introduced a deep learning algorithm designed for detecting LV systolic dysfunction using standard 12-lead ECGs [12]. However, there is no evidence yet to confirm that the AI-ECG model we have developed can truly improve the management of ALVSD in clinical practice.

A previous landmark randomized controlled trial (RCT) used AI-ECG in primary care to alert frontline physicians about patients at high risk for low LVEF [13]. The study found that in the intervention group, 2.1% of patients received a new diagnosis of low LVEF, compared to 1.6% in the control group. This difference was primarily driven by the subgroup of patients with positive AI-ECG results—14.5% in the control arm versus 19.5% in the intervention arm. This study highlighted the potential of applying AI-ECG in primary care. To date, no studies have specifically focused on hospitalized patients. Hospitalized patients are more likely to have low LVEF compared to outpatients, but they also receive more intensive care, which may reduce underdiagnosis. This piqued our interest in understanding whether AI-ECG could improve the detection rate of low LVEF in hospitalized patients. We plan to conduct an RCT involving non-cardiology inpatients to explore whether AI-ECG can still provide additional benefits in an inpatient setting.

## Methods

### AI-ECG model and high-risk classification

We employed a previously developed deep learning model that analyzes standard 12-lead ECGs to estimate the likelihood that a patient’s left ventricular ejection fraction (EF) is ≤ 50% [14]. Briefly, this model was trained on a large, retrospectively collected ECG dataset linked with corresponding echocardiographic EF measurements. The AI model was designed to generate continuous-valued predictions of EF.

To classify patients as “high risk,” we selected an optimal probability threshold based on receiver operating characteristic (ROC) analysis. Specifically, we examined sensitivity, specificity, and the area under the ROC curve (AUC) across a range of cutoffs. We chose a threshold that balanced sensitivity and specificity, while maintaining a clinically acceptable false-positive rate. Internal validation using a hold-out dataset demonstrated that at this threshold, the model achieved a sensitivity of 72.4% and a specificity of 89.1% for detecting EF ≤ 50%. Further details of the model’s architecture, training methodology, and validation can be found in our previous publication [14].

In the present study, whenever a patient’s ECG yielded a model probability score greater than or equal to the threshold, the system automatically flagged the result as “AI high risk.” Conversely, patients with scores below the threshold were labeled “AI low risk.” For patients in the intervention arm, the hospital information system generated an on-screen alert to notify clinicians of the high-risk status in real time; no such alert was provided for the control arm. Clinicians could then decide whether to pursue additional diagnostic evaluations (such as echocardiogram) based on both the AI classification and their clinical judgment.

### Trial design and ethical statement

This trial was registered with ClinicalTrials.gov (NCT05117970) and followed CONSORT-AI Extension checklist guidelines. The study received ethical approval from Tri-Service General Hospital's institutional review board (IRB) in Taipei, Taiwan (A202105120). Given that patient interaction was limited to Electronic Health Records (EHR) data collection, informed consent was obtained from attending physicians before trial initiation. This consent approach was justified by four principles: minimal patient risk (compliant with Taiwan Food and Drug Administration guidelines), universal hospital visitor inclusion rather than specific population targeting, maintenance of participants'rights and welfare through standard clinical care, and full disclosure of AI-ECG results during medical decision-making.

At a Taiwanese academic medical center, we conducted a RCT where attending physicians who provided informed consent were enrolled in the AI-ECG report system, while non-consenting physicians were excluded. Although patients were not directly enrolled as participants, we analyzed their EHR data to evaluate the AI-ECG intervention's effectiveness.

The study included patients who underwent at least one ECG examination between November 2022 and May 2023 in the inpatient department under the care of non-cardiologists. Our hospital follows international healthcare systems'ECG indications, primarily based on routine ward protocols and relevant clinical criteria, including: (1) routine ECG examinations for middle to advanced age patients requiring operation or hospitalization, (2) ECGs for patients with symptoms related to heart rhythm disturbances, chest pain, or suspected acute coronary syndrome, (3) ECGs for patients with existing or suspected cardiovascular diseases, (4) evaluation of bradycardia or tachycardia, (5) assessment of electrolyte imbalances and drug toxicity, and (6) monitoring of patients with implanted cardiac devices. Exclusion criteria were applied to patients under 18 years old or patients with LVEF of ≤ 50% within the previous 180 days. The physicians used the six ECG examination criteria according to clinical practice guidelines. These criteria were not recorded as independent data points for each participant. Therefore, they did not appear in the dataset. Our analysis focused solely on patients cared for by attending physicians who had provided informed consent.

### Randomization

At our hospital, each patient is assigned a unique 7-digit medical record number, from a pool of 10^7^ possible combinations. We implemented patient-level randomization rather than physician-level assignment to maximize follow-up retention and ensure consistent care throughout the 30-day study period. Before beginning the trial, we used simple random sampling to divide these 10 possible medical record numbers equally, allocating 5 numbers to the intervention group and 5 to the control group. This randomization scheme was established before patients received their medical record numbers, enabling pre-allocation of future patients to study groups based on their subsequently assigned medical record numbers.

### Data collection process

We divided our study population into high-risk and low-risk subgroups to evaluate the warning message's impact on medical care. For all patients who had at least one high-risk ECG (in both intervention and control groups), we started the follow-up period from the time of their first AI-identified high-risk ECG. This timing was applied consistently across both groups, even though control group patients did not receive warning messages. For patients whose ECGs showed no high-risk findings, the follow-up period began at the time of their first ECG examination.

For high-risk patients, we selected their first high-risk ECG as the index time, as this moment represented the earliest opportunity for medical intervention and potential outcome improvement. For low-risk patients, who by definition had no high-risk ECGs, we used their first ECG as the index time. While these different index time definitions created distinct high-risk and low-risk subgroups, this categorization was independent of the study's randomization process.

We extracted patient demographic and clinical data from our hospital information system, including sex, age, and pre-existing medical conditions identified using ICD-9 and ICD-10 codes: Diabetes Mellitus (DM: ICD-9 250.x; ICD-10 E08.x-E13.x), Hypertension (HTN: ICD-9 401.x-404.x; ICD-10 I10.x-I16.x), Hyperlipidemia (HLP: ICD-9 272.x; ICD-10 E78.x), Chronic Kidney Disease (CKD: ICD-9 585.x; ICD-10 N18.x), Acute Myocardial Infarction (AMI: ICD-9 410.x; ICD-10 I21.x), Stroke (STK: ICD-9 430.x-438.x; ICD-10 I60.x-I63.x), Coronary Artery Disease (CAD: ICD-9 410.x-414.x, 429.2; ICD-10 I20.x-I25.x), Heart Failure (HF: ICD-9 428.x; ICD-10 I50.x), Atrial Fibrillation (Afib: ICD-9 427.31; ICD-10 I48.x), and Chronic Obstructive Pulmonary Disease (COPD: ICD-9 490.x-496.x; ICD-10 J44.9).

### Primary and secondary endpoints

The primary outcome of this study was the incidence of newly diagnosed low EF (≤ 50%) within 30 days. Secondary outcomes included echocardiogram usage and 30-day all-cause mortality.

### Post-hoc analyses

During data examination, we observed variations in how echocardiograms were utilized among high-risk patients. To explore this pattern, we conducted an additional analysis examining the relationship between cardiology consultation rates, echocardiogram usage, and the detection of newly diagnosed low EF. Because cardiology consultations were not originally defined as a secondary endpoint in our protocol, we have designated these analyses as post-hoc.

### Sample size

We performed sample size estimation using a significance level of 0.05, a statistical power of 0.80, a sample size ratio in intervention and control groups of 1.0, a hypothetical proportion of controls with a primary endpoint of 0.03 [15] and a relative risk of 1.32 [13], and the minimum number in intervention and control group were both 6,032 per arm.

### Statistical analysis

Statistical analyses were performed using R version 3.4.4, with statistical significance defined as *p* < 0.05. Patient characteristics and ECG features were summarized using means with standard deviations or percentages where appropriate. Student's t-test evaluated differences in randomization and AI-ECG predictions, while chi-square test analyzed categorical variables. We utilized a Cox proportional hazard mixed effect model (R package"coxme"version 2.2–18.1) to compare primary and secondary endpoints between intervention and control groups, incorporating enrolled physicians as random effects. Treatment effects were expressed as hazard ratios (HRs) with 95% confidence intervals (95% CIs), and event cumulative incidence was visualized using Kaplan–Meier curves. In this study, we elected to use a Cox proportional hazards mixed-effects model, rather than a linear (or logistic) regression model, for several reasons. First, our primary outcome (newly diagnosed low EF) was measured as a time-to-event outcome, and some patients were censored if they were discharged, transferred, or lost to follow-up before day 30. Cox models naturally handle such right-censoring, whereas linear or logistic regression would not capture the timing of each event. Second, the use of a mixed-effects model allowed us to include attending physicians as a random effect to account for potential clustering of clinical decision-making. This better reflects real-world practice variations and prevents overestimating the precision of our estimates. Finally, a proportional hazards framework provides hazard ratios that describe how the instantaneous risk of a new low EF diagnosis changes over time—an approach well-suited to many clinical outcomes that may arise at varying points within a specified follow-up window. Subgroup analyses stratified by age, sex, and baseline comorbidities were performed, with subgroup effects assessed through interaction terms in the Cox model. All analyses beyond the primary endpoint were considered exploratory.

## Results

### Patient characteristics

**Fig. 1:**
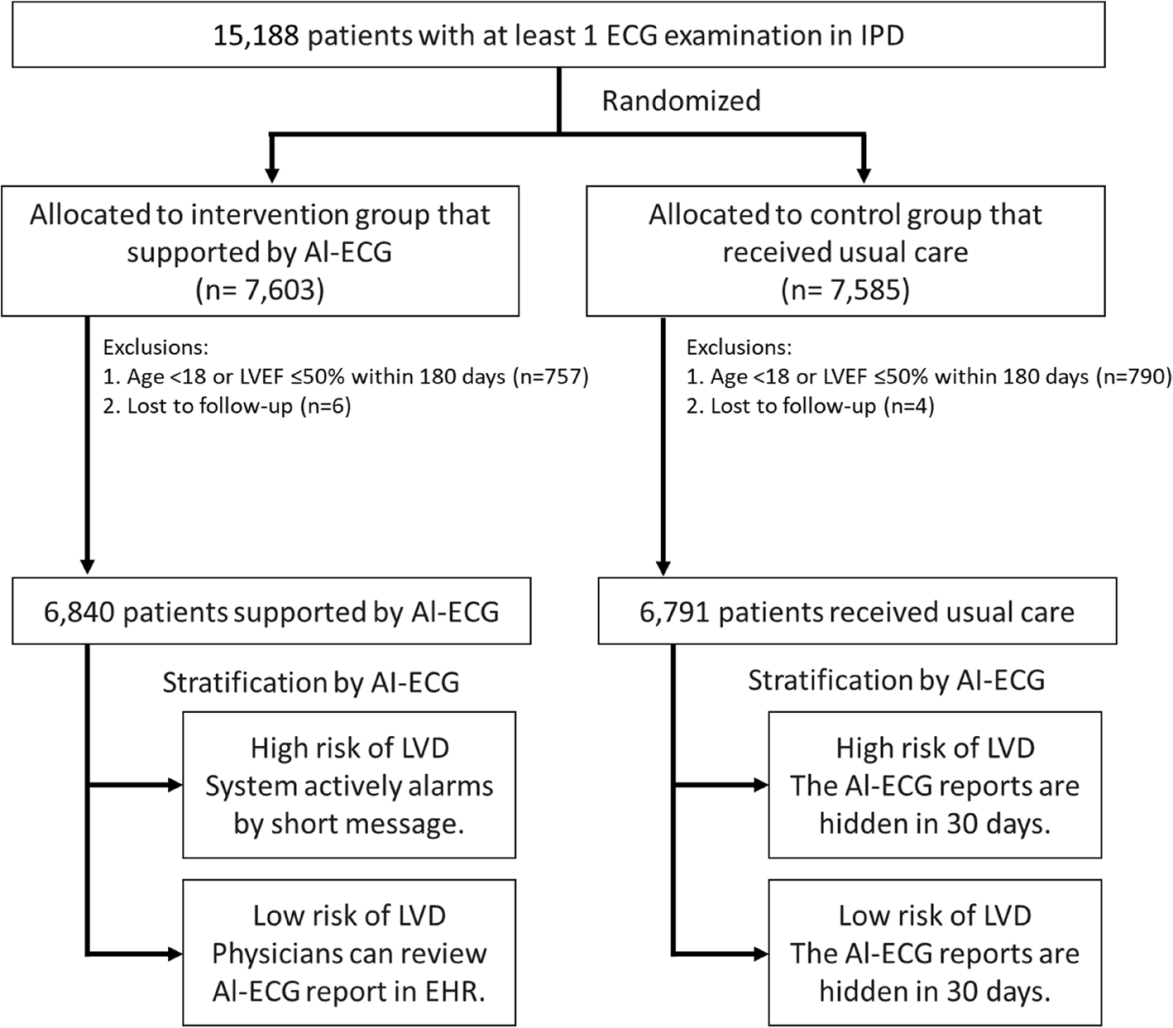
CONSORT-AI flow diagram. Abbreviations: IPD, inpatient department; ECG, electrocardiography; AI-ECG, artificial intelligence-enabled electrocardiogram for LVD (left ventricular dysfunction) stratification; and EHR, electronic health record

As depicted in Fig. 1, the final analysis encompassed 6,840 patients in the intervention group and 6,791 patients in the control group. Within these groups, the AI-ECG identified 629 patients (9.2%) as high-risk for LVD in the intervention group, and 583 patients (8.6%) in the control group. Physicians may receive AI-ECG alerts for high-risk patients in the intervention group and subsequently conduct thorough assessments of their current health status, arranging appropriate examination for them. The mean age of patients was 59.86 ± 17.38 years in the intervention group and 59.91 ± 17.22 years in the control group, with a nearly equal distribution of 45.4% male patients in both the intervention and control groups (Table 1). For more detailed comparisons between the intervention and control groups stratified by AI-ECG findings, please refer to Table S1.

**Table 1:** Patient characteristics stratified by randomization

	Control (*n* = 6791)	Intervention (*n* = 6840)	*p*-value
Stratification by AI-ECG			0.210
Low risk of LVD	6208(91.4%)	6211(90.8%)	
High risk of LVD	583(8.6%)	629(9.2%)	
Demographics			
GENDER			0.997
Female	3710(54.6%)	3737(54.6%)	
Male	3081(45.4%)	3103(45.4%)	
Age (mean ± SD)	59.91 ± 17.22	59.86 ± 17.38	0.859
Age group			0.799
< 65 y/o	3981(58.6%)	3971(58.1%)	
65–75 y/o	1540(22.7%)	1572(23.0%)	
≥ 75 y/o	1270(18.7%)	1297(19.0%)	
Comorbidities			
DM	1528(22.5%)	1493(21.8%)	0.344
HTN	2227(32.8%)	2281(33.3%)	0.491
CKD	1517(22.3%)	1507(22.0%)	0.667
HLP	2285(33.6%)	2274(33.2%)	0.619
AMI	132(1.9%)	142(2.1%)	0.582
STK	683(10.1%)	653(9.5%)	0.316
CAD	1241(18.3%)	1185(17.3%)	0.147
Afib	451(6.6%)	473(6.9%)	0.524
COPD	769(11.3%)	754(11.0%)	0.578

*Abbreviations*: SD Standard deviation, DM Diabetes mellitus, HTN Hypertension, CKD Chronic kidney disease, HLP Hyperlipidemia, AMI Acute myocardial infarction, STK Stroke, CAD Coronary artery disease, Afib Atrial fibrillation, COPD Chronic obstructive pulmonary disease

### Primary endpoint analysis

**Fig. 2:**
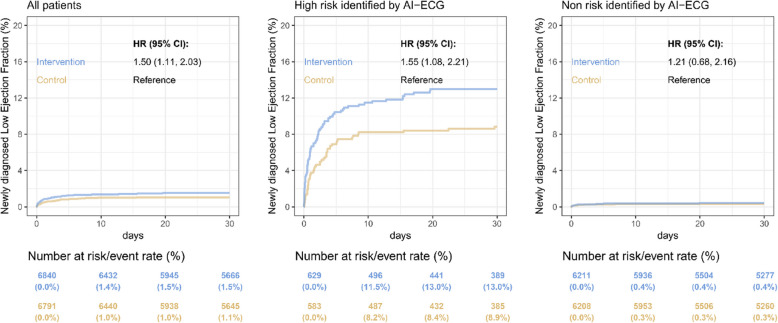
AI-ECG intervention for primary endpoint. Kaplan–Meier curve analysis of new-onset low ejection fraction at 30 days. The p for interaction between risk stratification of AI-ECG and intervention/control was 0.023

**Fig. 3:**
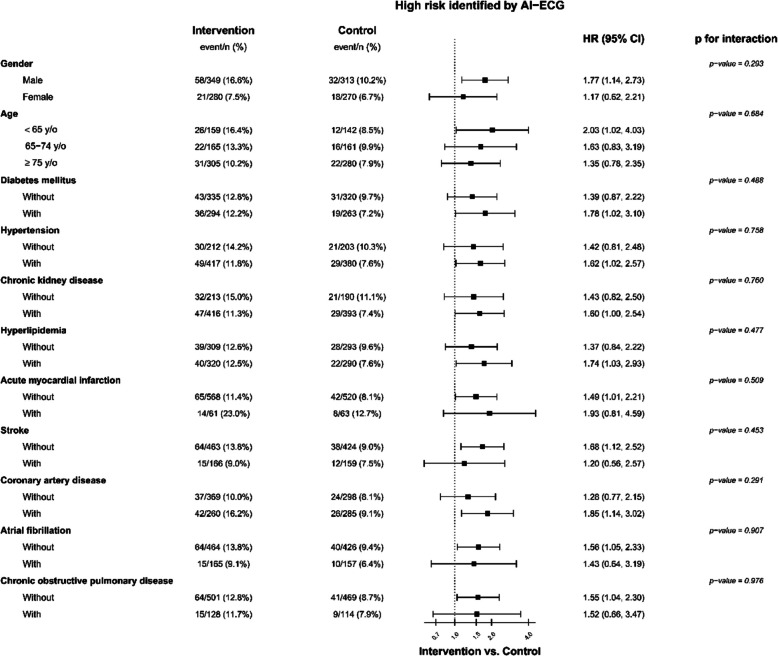
The forest plot of AI-ECG intervention for primary endpoint. Subgroup analysis in patients with a high risk of low ejection fraction identified by AI-ECG for new-onset low ejection fraction within 30 days. The p for interaction was two-sided, with no adjustment for multiple comparisons

Figure 2 illustrates that in the overall population, there was a significant difference in the occurrence of newly diagnosed low EF within 30 days between the two groups, with 1.5% in the intervention group compared to 1.1% in the control group. The hazard ratio (HR) was 1.50, with a 95% confidence interval (CI) of 1.11–2.03. The active warning message in the intervention group increased the detection of newly diagnosed low EF by 55% (HR: 1.55 and 95% CI: 1.08–2.21). However, the opportunity to review AI-ECG reports had limited impact on the AI-defined low-risk population (HR: 1.21 and 95% CI: 0.67–2.16). The effect of the intervention on newly diagnosed low EF was largely consistent across various subgroups (Fig. 3). Interestingly, there was a nonsignificant trend indicating more pronounced benefits among male or younger patients, as well as those with diabetes mellitus, hypertension, chronic kidney disease, hyperlipidemia, and coronary artery disease. On the other hand, the benefits were also as evident in patients without acute myocardial infarction, stroke, atrial fibrillation, or chronic obstructive pulmonary disease.

### Secondary endpoints analysis

**Fig. 4:**
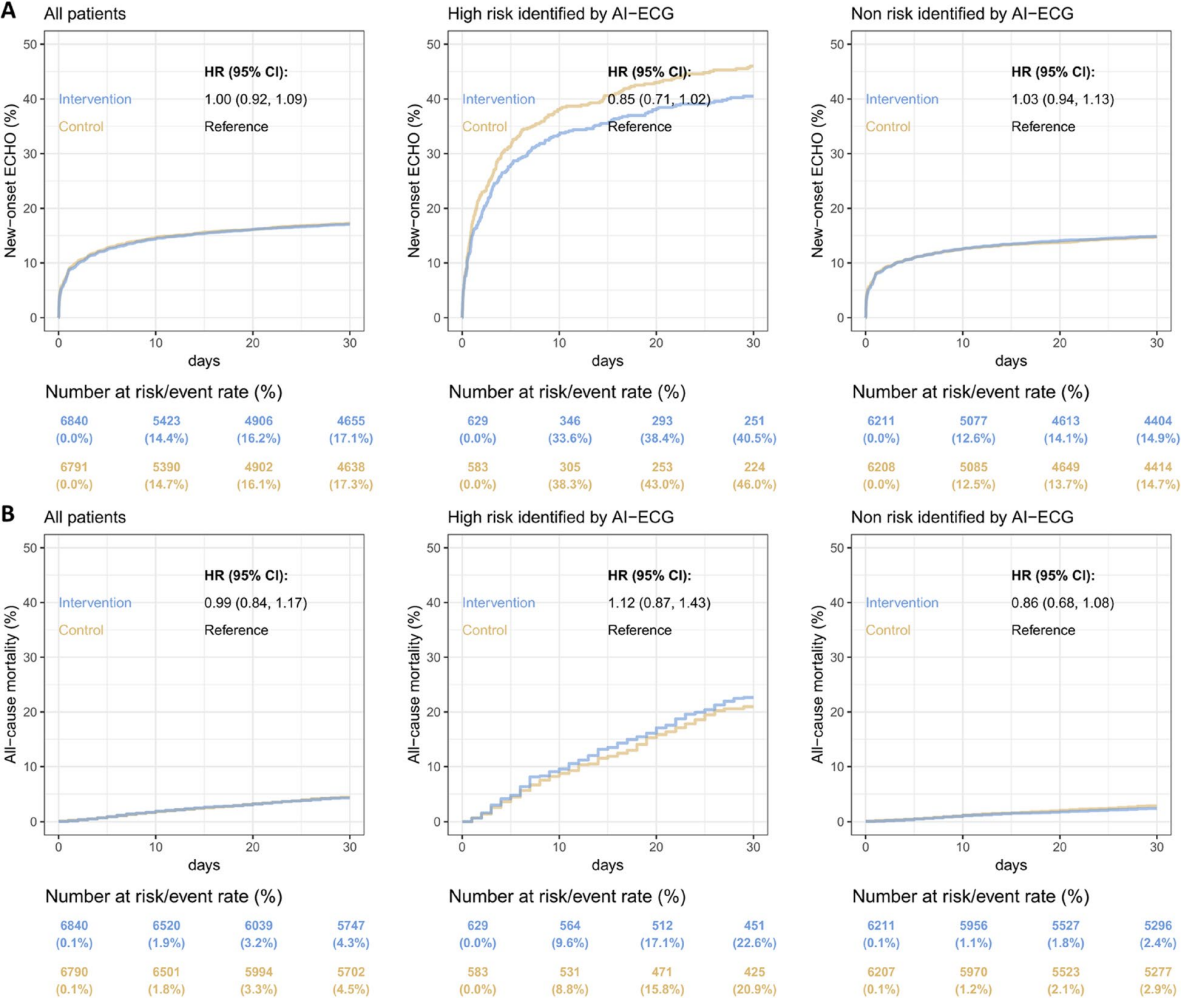
AI-ECG intervention for secondary endpoint. Kaplan–Meier curve analysis of underwent echocardiogram and all-cause mortality at 30 days

Across the entire population, there were no significant differences observed in the proportions of patients undergoing echocardiogram between the control and intervention groups (17.3% in the control group compared to 17.1% in the intervention group, HR: 1.00 (95% CI: 0.92–1.09)). Similarly, in terms of all-cause mortality, there were no statistically significant distinctions observed between the control and intervention groups across the entire population (4.5% in the control group compared to 4.3% in the intervention group, HR 1.00 (0.84–1.17)). In the analysis of secondary outcomes within the AI-defined high-risk or low-risk populations, there was also no difference between the intervention group and the control group. (Fig. 4). Notably, although the proportion of echocardiogram showed no statistically significant difference across the entire population or within the AI-defined high-risk or low-risk groups, we observed that the proportion appeared slightly higher in the control group.

### Comparison the effectiveness of intervention and control groups

**Fig. 5:**
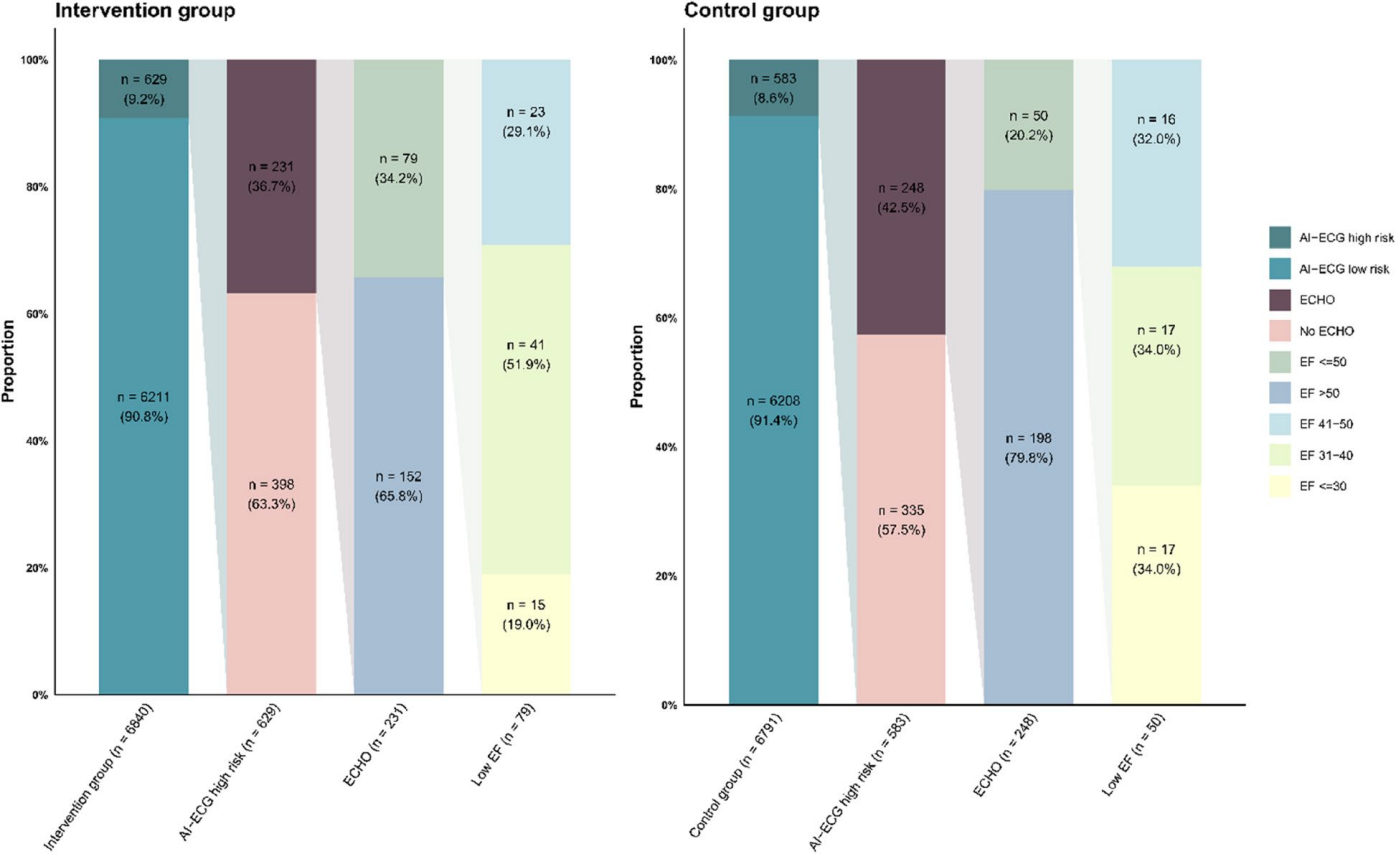
The effectiveness for low ejection fraction diagnosis in AI-ECG-identified high-risk between Intervention and Control groups. Stacked bar plots display the proportion and percentage of events by each condition

Of the participants in the Intervention group (*n* = 6840), 9.2% (629 individuals) found to be high-risk group by AI-ECG. Of those in the high-risk group, 231 participants were arranged for echocardiograms by physicians. Subsequently, 34.2% (79 of 231) of individuals been diagnosed with low EF. Compared to participants in the Control group who found to be high-risk group recommended echocardiogram, the AI-support group exhibited a higher positive predictive value for low EF identification (34.2% [79 of 231] vs 20.2% [50 of 248]; *p* < 0.001) (Fig. 5). Moreover, in Figure S1, we also noticed that when low EF is categorized by different severity levels, the real impact is seen primarily in the increased detection of moderate low EF. In the intervention group increased the detection of newly diagnosed low EF between 31 to 40 by 132% (HR: 2.32 and 95% CI: 1.32–4.09). Alternatively, In the intervention group increased the detection of newly diagnosed low EF between 31 to 50 by 76% (HR: 1.76 and 95% CI: 1.17–2.64). However, there was no significant difference in the detection of mild (EF between 41 to 50) or severe (EF < = 30) cases.

### Post-hoc analysis

It's worth noting that although more low EF patients were identified in the Intervention group, the proportion of patients receiving echocardiogram rates was not higher. Given our observation of higher cardiology consultation rates among high-risk patients, we delved deeper into the mechanism behind the increased detection of low EF cases. Table 2 reveals a significant difference in cardiology consultation rates between the intervention and control groups for all patients in the high-risk category. The intervention significantly increased cardiology consultation rates among high-risk patients (29.3% vs. 23.5%, *p* = 0.027). This effect was not observed in low-risk patients (5.1% vs. 4.6%, *p* = 0.279). Interestingly, the overall echocardiogram rates among patients who received cardiology consultations were similar between the intervention and control groups for both high-risk (52.2% vs. 49.6%, *p* = 0.736) and low-risk patients (39.2% vs. 38.3%, *p* = 0.897). However, among high-risk patients who did not receive cardiology consultations, the control group had a higher rate of echocardiograms compared to the intervention group (40.4% vs. 30.3%, *p* = 0.002). As our results in Table 2 demonstrate, physicians in the intervention group may have felt more confident in the AI-ECG results, leading to more selective use of echocardiograms, while those in the control group, lacking this AI support, might have been more inclined to order echocardiograms as a precautionary measure for patients they identified as high-risk through traditional methods. We also analyzed EF distributions among high-risk patients who received both cardiology consultations and echocardiograms. No significant differences were found between the intervention and control groups (*p* = 0.157). In Table S2, A notable finding was that in the intervention group, high-risk patients with cardiology consultations were significantly more likely to receive an echocardiogram compared to those without consultations (52.2% vs. 30.3%, *p* < 0.001). This difference was not observed in the control group (49.6% vs. 40.4%, *p* = 0.068). These results suggest that the intervention was effective in increasing cardiology consultations for high-risk patients, which may have led to more targeted use of echocardiograms. This approach could potentially improve the detection of low EF cases, even though the overall proportion of echocardiograms performed was not significantly different between the groups.

**Table 2:** Post-hoc analysis for mechanism of more low EF findings

	High risk			Low risk		
	Control	Intervention	*p*-value	Control	Intervention	*p*-value
All patients	*n* = 583	*n* = 629	0.027	*n* = 6208	*n* = 6211	0.279
Without cardiologist consultation	446(76.5%)	445(70.7%)		5921(95.4%)	5897(94.9%)	
With cardiologist consultation	137(23.5%)	184(29.3%)		287(4.6%)	314(5.1%)	
Patients with cardiologist consultation	*n* = 137	*n* = 184	0.736	*n* = 287	*n* = 314	0.897
Without echocardiogram	69(50.4%)	88(47.8%)		177(61.7%)	191(60.8%)	
With echocardiogram	68(49.6%)	96(52.2%)		110(38.3%)	123(39.2%)	
Patients without cardiologist consultation	*n* = 446	*n* = 445	0.002	*n* = 5921	*n* = 5897	0.938
Without echocardiogram	266(59.6%)	310(69.7%)		5139(86.8%)	5122(86.9%)	
With echocardiogram	180(40.4%)	135(30.3%)		782(13.2%)	775(13.1%)	
Patients with cardiologist consultation and echocardiogram	*n* = 68	*n* = 96	0.157	*n* = 110	*n* = 123	0.281
EF > 50%	51(75.0%)	60(62.5%)		101(91.8%)	107(87.0%)	
EF 31–50%	10(14.7%)	26(27.1%)		9(8.2%)	14(11.4%)	
EF ≤ 30%	7(10.3%)	10(10.4%)		0(0.0%)	2(1.6%)	
Patients without cardiologist consultation with echocardiogram	*n* = 180	*n* = 135	0.002	*n* = 782	*n* = 775	0.797
EF > 50%	147(81.7%)	92(68.1%)		770(98.5%)	766(98.9%)	
EF 31–50%	23(12.8%)	38(28.2%)		11(1.4%)	8(1.0%)	
EF ≤ 30%	10(5.5%)	5(3.7%)		1(0.1%)	1(0.1%)	

*Abbreviations*: *EF* Ejection fraction

## Discussion

While numerous AI algorithms have been developed and validated for medical applications, only a handful have undergone prospective evaluation in RCTs. The current study stands as one of the pioneering RCTs aimed at assessing the efficacy of an AI-powered clinical decision support tool in everyday clinical practice. The trial highlights its success in enhancing the detection of low EF, a condition often lacking noticeable symptoms and frequently underdiagnosed in its early stages. By establishing an information technology infrastructure that automatically analyzes ECGs and promptly communicates AI-generated results to healthcare providers, this novel tool offers a promising avenue for early identification and management of low EF. This, in turn, holds the potential to mitigate the disease burden and reduce mortality in larger populations. Nonetheless, it is crucial to underline that further investigations are imperative to ascertain the cost-effectiveness of this approach and gauge the subsequent clinical impact of these newly established diagnoses.

The addition of AI to ECG can enhance the value of this widely used, cost-effective test, but its impact depends on several factors. It's most useful in populations with higher rates of missed or delayed diagnoses, as asymptomatic left ventricular dysfunction affects 3%−6% of the general population and can be treatable if detected early [16]. In 2019, Attia's algorithm achieved an AUC of 0.93 for detecting patients with EF ≤ 35% [16]. Our research has yielded similar accuracy [12]. Adedinsewo et al. found an algorithm with an AUC of 0.89 for identifying patients with EF ≤ 35% and an AUC of 0.85 for EF < 50% [17], outperforming the NT-proBNP test [18]. Kashou's algorithm showed an AUC of 0.93 and sensitivity and specificity over 85% for EF ≤ 35% and an AUC of 0.97 for EF ≤ 40% in a community-based cohort [19]. These results highlight the potential of AI-enhanced ECGs in diagnosing asymptomatic left ventricular dysfunction, especially in high-risk populations [19].

The magnitude of impact in our study is also contingent upon the responsiveness of healthcare professionals to AI-driven recommendations. In a prior investigation, the intervention led to a discernible escalation in the utilization of echocardiograms, with rates rising from 38.1% to 49.6% [13]. Conversely, our study did not yield a statistically significant increase in utilization either in AI-high or low risk group. However, there is scope for enhancement in this regard. Typical symptoms in patients with low LVEF may include dyspnea, orthopnea, paroxysmal nocturnal dyspnea, fatigue, and ankle swelling. Evaluation can involve measuring natriuretic peptides, conducting electrocardiography, and performing chest X-rays [20]. Echocardiogram imaging is too resource-intensive for screening unselected populations. This aligns with our earlier post-hoc findings, leading to more selective use of echocardiograms, while those in the control group, lacking this AI support, might have been more inclined to order echocardiograms as a precautionary measure for patients they identified as high-risk through traditional methods. A more affordable and widely accessible test, such as the AI-ECG, could help identify high-risk patients who should then undergo further evaluation with echocardiography [21]. Echocardiogram is a golden standard to diagnose diverse cardiac diseases, such as low LVEF, valvular heart diseases, structure heart diseases, etc. [22]. Physicians may arrange more echocardiograms for patients with a high likelihood of low LVEF after thorough assessments. This approach would reduce the need to schedule testing for low-risk groups. Therefore, the AI-ECG actually prompted physicians to carefully assess patient conditions, leading to more patients with high likelihood of low LVEF receiving echocardiogram (as show in Table S2). Our investigation revealed that employing an AI-ECG algorithm led to a higher detection rate of low EF in the AI-supported group (34.2% in the intervention arm, compared to 20.2% in the conventional group). This suggests a modest but statistically significant improvement linked to the use of AI-ECG.

The impact of the tool also depends on the cut-point selected to trigger clinician action. In the current study, we selected the cut-point used in the initial derivation study that optimized the sensitivity and specificity equally. Only 9.2% of the patients had a positive AI-ECG result, and the overall use of echocardiogram was not different between intervention and control groups, providing some reassurance that even this relatively sensitive threshold would not increase overall healthcare utilization. In clinical practice, healthcare providers routinely integrate diverse information sources, including clinical decision support (CDS) guidance, to inform their decision-making processes [23]. Within this framework, assistive CDS often necessitates that clinicians critically evaluate"black-box"CDS recommendations alongside their own clinical judgment. For instance, when assessing the risk of surgical complications, clinicians must conduct an independent evaluation that encompasses a patient's medical history, physical examination, laboratory findings, and additional diagnostic tests, in addition to incorporating CDS insights [24]. This dual evaluation process, integral to the clinical workflow, can introduce additional time demands and potential decision delays, particularly when there is misalignment between the clinician's judgment and the CDS recommendations. Such discrepancies may elevate the risk to patient safety [25]. In addition to our pre-specified primary (new diagnoses of EF ≤ 50%) and secondary outcomes (echocardiogram usage and mortality), we performed a post-hoc analysis to clarify how cardiology consultations may have influenced the detection of newly diagnosed low EF. This analysis was introduced because, although we noted a higher detection rate of low EF in the intervention arm, the overall echocardiogram usage did not differ markedly between groups. By examining cardiology consultation as an additional factor, we aimed to elucidate the pathway through which AI alerts might improve diagnostic precision. It is important to acknowledge that this consultation analysis was not part of our original trial design. As a result, the findings from this post-hoc investigation should be interpreted with caution and viewed primarily as hypothesis-generating. Future prospective trials could consider including consultation metrics as a formally pre-specified endpoint to validate our observations in a more controlled and hypothesis-driven manner.

Previous investigations into AI-enabled ECG alerts have largely focused on primary care or community-based cohorts, where underdiagnosis of low EF may be more pronounced. For instance, Yao et al. conducted a pragmatic trial in outpatient settings, demonstrating that an AI-ECG alert significantly increased echocardiogram orders and new low EF diagnoses [13]. Similar results were reported by Attia et al. in a population-based study designed to screen for asymptomatic left ventricular systolic dysfunction [26]. In these outpatient contexts, clinicians often have fewer immediate diagnostic resources available and may rely on screening tools to help identify patients requiring further cardiac evaluation. By contrast, our study was conducted in an inpatient environment where patients receive more intensive monitoring from multiple specialists, which can inherently reduce missed diagnoses of cardiovascular dysfunction. Consequently, the effect of our AI-based tool manifested primarily in improving the efficiency or positive predictive value (PPV) of diagnostic testing, rather than simply increasing the frequency of echocardiograms. While the overall echocardiogram utilization did not differ substantially between the intervention and control groups, patients flagged as high risk by AI in the intervention arm had a higher yield of truly low EF. These findings highlight that, in a setting already characterized by robust diagnostic vigilance, an AI alert can guide more judicious use of echocardiogram and consultation—enhancing diagnostic precision without imposing a heavier testing burden. Future investigations conducted across various inpatient and outpatient settings may help delineate how local practice patterns, resource availability, and baseline diagnostic thresholds influence the performance and clinical impact of AI-ECG alerts.

Our study has several limitations to consider. First, it was conducted at a single academic center, which may limit the generalizability of the findings to other healthcare settings with different patient demographics or resource availability. Second, while our data were collected pragmatically from electronic health records (EHR), there remains a possibility of missing or incomplete documentation. Third, although we used a Cox proportional hazards mixed-effects model to account for physician-level variability, other unmeasured confounders could still influence the results. Additionally, there is a noteworthy discrepancy between our initial registry entry (NCT05117970) and the final study design. The original protocol specified a primary outcome of EF ≤ 35% over 90 days and a target sample size of 84,000, predicated on a multi-center approach with a longer enrollment period. During the early implementation phase, however, we determined that EF ≤ 50% was more clinically pertinent for inpatients, as it includes those with mildly reduced EF. We also found that a 30-day follow-up window accurately captured newly diagnosed low EF in the inpatient setting without prolonging the observation period unnecessarily. Consequently, we refined our inclusion criteria, reduced the scope to a single center, and updated our trial registry in October 2024 to reflect these parameters, acknowledging that this timing was later than ideal. Although any post-hoc modification to a registered study can raise concerns about protocol adherence, we have disclosed all changes and their rationale to ensure transparency. Finally, information regarding patient symptoms and emergency department visits was not collected in this study. Despite these limitations, our findings highlight the potential of an AI-enabled ECG platform to improve early detection of low EF in hospitalized patients under non-cardiologist care. Future studies involving multiple centers, longer follow-up periods, and broader patient populations would help further validate these results and explore their generalizability to other clinical environments.

## Conclusions

The utilization of an AI algorithm on existing ECGs facilitated the early detection of low EF in a substantial patient cohort managed within everyday inpatient settings. Given that ECG is a cost-effective test commonly conducted for various medical purposes, this algorithm has the potential to enhance the early diagnosis and management of a condition that is frequently asymptomatic but is amenable to effective treatments. Notably, this study found that the diagnosis rate of low LVEF improved without an increase in echocardiogram utilization, likely due to careful evaluation after consulting cardiologists. This highlights the differences in the application of AI-ECG in inpatient settings compared to primary care. We recommend future research to broadly investigate the effectiveness of AI-ECG across different populations.

## Supplementary Information

Additional file 1: Figures S1. FigS1- [AI-ECG intervention for primary endpoint among different cut point]. Tables S1-S2. TableS1- [Patient characteristics stratified by AI-ECG]. TableS2- [Post-hoc analysis for high risk subgroup]

## Data Availability

No datasets were generated or analysed during the current study.

## Abbreviations

EF: Ejection fraction
ECG: Electrocardiogram
AI: Artificial intelligence
HR: Hazard ratios
CI: Confidence intervals
ALVSD: Asymptomatic left ventricular systolic dysfunction
LVEF: Left ventricular ejection fraction
RCT: Randomized controlled trial
ROC: Receiver operating characteristic
AUC: Area under the ROC curve
IRB: Institutional review board
EHR: Electronic health records
CDS: Clinical decision support

## References

[1] Mozaffarian D, et al. Heart disease and stroke statistics–2015 update: a report from the American Heart Association. Circulation. 2015;131:e29-322. 10.1161/CIR.0000000000000152. 25520374 10.1161/CIR.0000000000000152

[2] Ponikowski P, et al. 2016 ESC Guidelines for the diagnosis and treatment of acute and chronic heart failure: the task force for the diagnosis and treatment of acute and chronic heart failure of the European Society of Cardiology (ESC)Developed with the special contribution of the Heart Failure Association (HFA) of the ESC. Eur Heart J. 2016;37:2129–200. 10.1093/eurheartj/ehw128. 27206819 10.1093/eurheartj/ehw128

[3] Yancy CW, et al. 2017 ACC/AHA/HFSA focused update of the 2013 ACCF/AHA guideline for the management of heart failure: a report of the American College of Cardiology/American Heart Association Task Force on clinical practice guidelines and the Heart Failure Society of America. Circulation. 2017;136:e137–61. 10.1161/CIR.0000000000000509. 28455343 10.1161/CIR.0000000000000509

[4] Yusuf S, Pitt B, Davis CE, Hood WB Jr, Cohn JN. Effect of enalapril on mortality and the development of heart failure in asymptomatic patients with reduced left ventricular ejection fractions. N Engl J Med. 1992;327:685–91. 10.1056/NEJM199209033271003. 1463530 10.1056/NEJM199209033271003

[5] Pfeffer MA, et al. Effect of captopril on mortality and morbidity in patients with left ventricular dysfunction after myocardial infarction. Results of the survival and ventricular enlargement trial. The SAVE investigators. N Engl J Med. 1992;327:669–77. 10.1056/NEJM199209033271001. 1386652 10.1056/NEJM199209033271001

[6] Echouffo-Tcheugui JB, Erqou S, Butler J, Yancy CW, Fonarow GC. Assessing the risk of progression from asymptomatic left ventricular dysfunction to overt heart failure: a systematic overview and meta-analysis. JACC: Heart Fail. 2016;4(4):237–48. 26682794 10.1016/j.jchf.2015.09.015

[7] Galasko GI, Barnes SC, Collinson P, Lahiri A, Senior R. What is the most cost-effective strategy to screen for left ventricular systolic dysfunction: natriuretic peptides, the electrocardiogram, hand-held echocardiography, traditional echocardiography, or their combination? Eur Heart J. 2006;27:193–200. 16267076 10.1093/eurheartj/ehi559

[8] Nielsen O, et al. Value of BNP to estimate cardiac risk in patients on cardioactive treatment in primary care. Eur J Heart Fail. 2007;9:1178–85. 18062902 10.1016/j.ejheart.2007.10.004

[9] Nielsen OW, McDonagh TA, Robb SD, Dargie HJ. Retrospective analysis of thecost-effectiveness of using plasmabrain natriuretic peptide inscreening for left ventricularsystolic dysfunction in the general population. J Am Coll Cardiol. 2003;41:113–20. 12570953 10.1016/s0735-1097(02)02625-6

[10] Goudie BM, et al. Screening for left ventricular systolic dysfunction using GP-reported ECGs. Br J Gen Pract. 2007;57:191–5. 17359605 PMC2042566

[11] Reichlin T, et al. Advanced ECG in 2016: is there more than just a tracing? Swiss Med Wkly. 2016;146:w14303–w14303. 27124801 10.4414/smw.2016.14303

[12] Chen H-Y, et al. Artificial intelligence-enabled electrocardiography predicts left ventricular dysfunction and future cardiovascular outcomes: a retrospective analysis. J Pers Med. 2022;12:455. 35330455 10.3390/jpm12030455 PMC8950054

[13] Yao X, et al. Artificial intelligence–enabled electrocardiograms for identification of patients with low ejection fraction: a pragmatic, randomized clinical trial. Nat Med. 2021;27:815–9. 10.1038/s41591-021-01335-4. 33958795 10.1038/s41591-021-01335-4

[14] Chen HY, et al. Artificial intelligence-enabled electrocardiogram predicted left ventricle diameter as an independent risk factor of long-term cardiovascular outcome in patients with normal ejection fraction. Front Med (Lausanne). 2022;9:870523. 10.3389/fmed.2022.870523. 35479951 10.3389/fmed.2022.870523 PMC9035739

[15] Wang TJ, et al. Natural history of asymptomatic left ventricular systolic dysfunction in the community. Circulation. 2003;108:977–82. 10.1161/01.Cir.0000085166.44904.79. 12912813 10.1161/01.CIR.0000085166.44904.79

[16] Attia ZI, et al. Screening for cardiac contractile dysfunction using an artificial intelligence–enabled electrocardiogram. Nat Med. 2019;25:70–4. 30617318 10.1038/s41591-018-0240-2

[17] Adedinsewo D, et al. Artificial intelligence-enabled ECG algorithm to identify patients with left ventricular systolic dysfunction presenting to the emergency department with dyspnea. Circ Arrhythm Electrophysiol. 2020;13:e008437. 32986471 10.1161/CIRCEP.120.008437

[18] Haq KT, Howell SJ, Tereshchenko LG. Applying artificial intelligence to ECG analysis: promise of a better future. Am Heart Assoc. 2020;13:e009111. 10.1161/CIRCEP.120.009111 32809878

[19] Kashou AH, et al. Artificial intelligence–augmented electrocardiogram detection of left ventricular systolic dysfunction in the general population. Mayo Clin Proc. Elsevier; 2021. 10.1016/j.mayocp.2021.02.029 PMC9904428 34120755

[20] Murphy SP, Ibrahim NE, Januzzi JL Jr. Heart failure with reduced ejection fraction: a review. JAMA. 2020;324:488–504. 10.1001/jama.2020.10262. 32749493 10.1001/jama.2020.10262

[21] Wang TJ, Levy D, Benjamin EJ, Vasan RS. The epidemiology of asymptomatic left ventricular systolic dysfunction: implications for screening. Ann Intern Med. 2003;138:907–16. 12779301 10.7326/0003-4819-138-11-200306030-00012

[22] Mertens L, Friedberg MK. The gold standard for noninvasive imaging in congenital heart disease: echocardiography. Curr Opin Cardiol. 2009;24:119–24. 10.1097/HCO.0b013e328323d86f. 19225295 10.1097/HCO.0b013e328323d86f

[23] van Baalen S, Boon M, Verhoef P. From clinical decision support to clinical reasoning support systems. J Eval Clin Pract. 2021;27:520–8. 33554432 10.1111/jep.13541 PMC8248191

[24] Brennan M, et al. Comparing clinical judgment with the MySurgeryRisk algorithm for preoperative risk assessment: a pilot usability study. Surgery. 2019;165:1035–45. 30792011 10.1016/j.surg.2019.01.002 PMC6502657

[25] Lyell D, et al. Automation bias in electronic prescribing. BMC Med Inform Decis Mak. 2017;17:1–10. 28302112 10.1186/s12911-017-0425-5 PMC5356416

[26] Attia ZI, et al. Screening for cardiac contractile dysfunction using an artificial intelligence–enabled electrocardiogram. Nat Med. 2019;25:70–4. 10.1038/s41591-018-0240-2. 30617318 10.1038/s41591-018-0240-2

