# Supplementary material for: Artificial intelligence-assisted diagnosis and prognostication in low ejection fraction using electrocardiograms in inpatient department: a pragmatic randomized controlled trial
Source: BMC Med. 2025 Jun 9;23:342. doi: 10.1186/s12916-025-04190-z (PMC12147261; doi:10.1186/s12916-025-04190-z)
Supplement: Supplementary file 1 — Additional file 1: Figures S1. FigS1- [AI-ECG intervention for primary endpoint among different cut point]. Tables S1-S2. TableS1- [Patient characteristics stratified by AI-ECG]. TableS2- [Post-hoc analysis for high risk subgroup] [file 12916_2025_4190_MOESM1_ESM.docx]

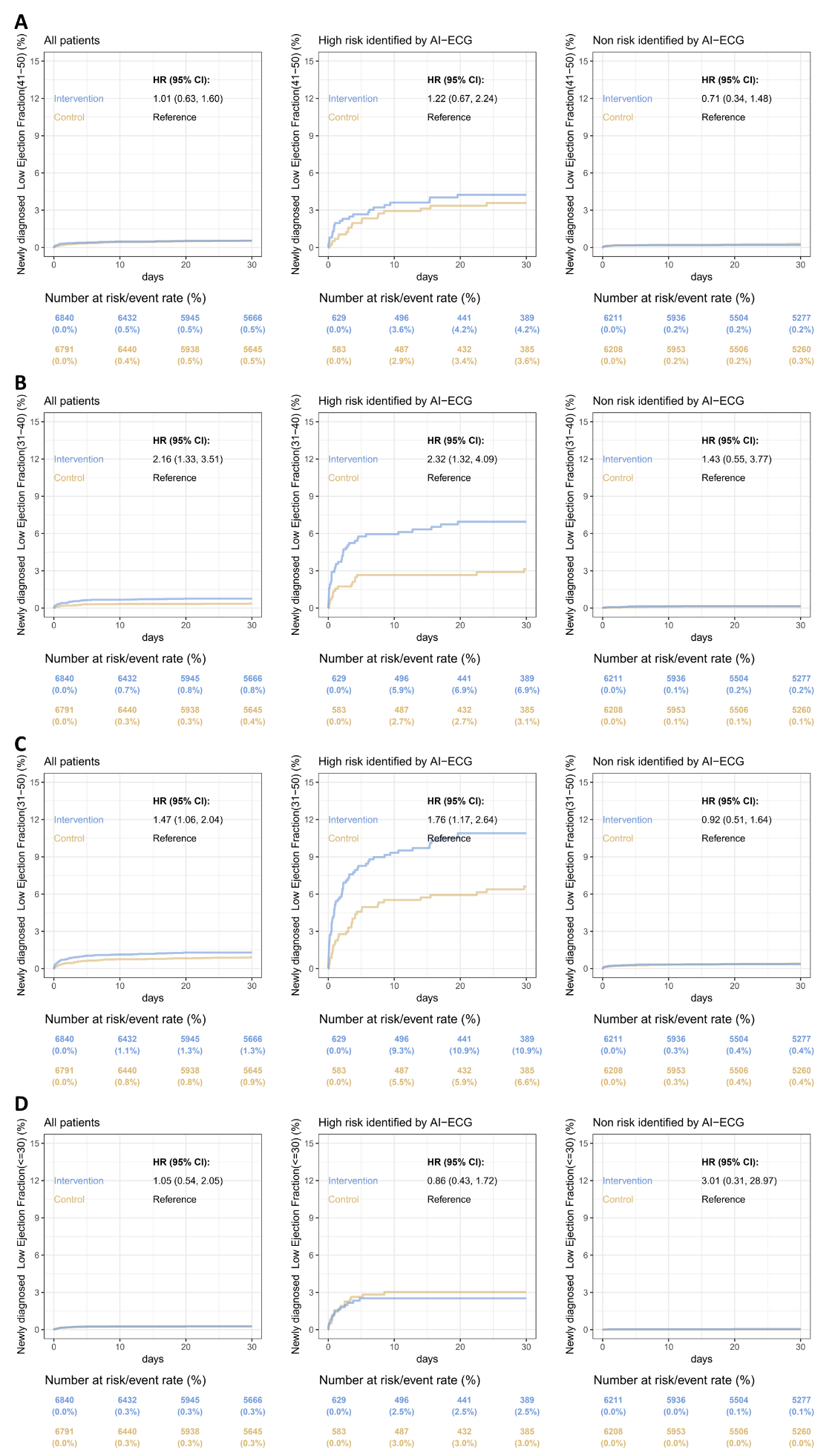


**Supplementary Figure 1.** **AI-ECG intervention for primary endpoint among different cut point.** Kaplan-Meier curve analysis of new-onset low ejection fraction at 30 days. (A) low ejection fraction between 41 to 50, (B) low ejection fraction between 31 to 40, (C) low ejection fraction between 31 to 50, (D) low ejection fraction less than 30.

**Supplementary Table 1 |** **Patient characteristics stratified by AI-ECG.**

|  | High risk | |  | Low risk | |  |
| --- | --- | --- | --- | --- | --- | --- |
|  | **Control** | **Intervention** | p-value | **Control** | **Intervention** | p-value |
| **Demographics** |  |  |  |  |  |  |
| GENDER |  |  | 0.530 |  |  | 0.782 |
| female | 270(46.3%) | 280(44.5%) |  | 3440(55.4%) | 3457(55.7%) |  |
| male | 313(53.7%) | 349(55.5%) |  | 2768(44.6%) | 2754(44.3%) |  |
| Age (mean ± SD) | 73.44±14.26 | 73.35±14.44 | 0.914 | 58.64±16.92 | 58.49±17.06 | 0.626 |
| Age group |  |  | 0.847 |  |  | 0.828 |
| < 65 y/o | 142(24.4%) | 159(25.3%) |  | 3839(61.8%) | 3812(61.4%) |  |
| 65-75 y/o | 161(27.6%) | 165(26.2%) |  | 1379(22.2%) | 1407(22.7%) |  |
| ≥ 75 y/o | 280(48.0%) | 305(48.5%) |  | 990(15.9%) | 992(16.0%) |  |
| **Comorbidities** |  |  |  |  |  |  |
| DM | 263(45.1%) | 294(46.7%) | 0.570 | 1265(20.4%) | 1199(19.3%) | 0.134 |
| HTN | 380(65.2%) | 417(66.3%) | 0.683 | 1847(29.8%) | 1864(30.0%) | 0.752 |
| CKD | 393(67.4%) | 416(66.1%) | 0.638 | 1124(18.1%) | 1091(17.6%) | 0.432 |
| HLP | 290(49.7%) | 320(50.9%) | 0.694 | 1995(32.1%) | 1954(31.5%) | 0.419 |
| AMI | 63(10.8%) | 61(9.7%) | 0.525 | 69(1.1%) | 81(1.3%) | 0.326 |
| STK | 159(27.3%) | 166(26.4%) | 0.729 | 524(8.4%) | 487(7.8%) | 0.222 |
| CAD | 285(48.9%) | 260(41.3%) | 0.008 | 956(15.4%) | 925(14.9%) | 0.431 |
| Afib | 157(26.9%) | 165(26.2%) | 0.784 | 294(4.7%) | 308(5.0%) | 0.563 |
| COPD | 114(19.6%) | 128(20.3%) | 0.729 | 655(10.6%) | 626(10.1%) | 0.387 |

Abbreviations: SD, standard deviation; DM: diabetes mellitus; HTN: hypertension; HLP: hyperlipidemia; CKD: chronic kidney disease; AMI: acute myocardial infarction; STK: stroke, CAD: coronary artery disease; HF: heart failure; Afib: atrial fibrillation; COPD: chronic obstructive pulmonary disease.

**Supplementary Table 2 |** **Post-hoc analysis for high risk subgroup**

|  | **Intervention** | |  | **Control** | |  |
| --- | --- | --- | --- | --- | --- | --- |
|  | **without cardiologist consultation** | **with cardiologist consultation** | p-value | **without cardiologist consultation** | **with cardiologist consultation** | p-value |
| **All high-risk patients** | n = 445 | n = 184 | <0.001 | n = 466 | n = 137 | 0.068 |
| Without echocardiogram | 310(69.7%) | 88(47.8%) |  | 266(59.6%) | 69(50.4%) |  |
| With echocardiogram | 135(30.3%) | 96(52.2%) |  | 180(40.4%) | 68(49.6%) |  |
| **Patients with echocardiogram** | n = 135 | n = 96 | 0.123 | n = 180 | n = 68 | 0.362 |
| EF >50% | 92(68.1%) | 60(62.5%) |  | 147(81.7%) | 51(75.0%) |  |
| EF 31-50% | 38(28.2%) | 26(27.1%) |  | 23(12.8%) | 10(14.7%) |  |
| EF ≤30% | 5(3.7%) | 10(10.4%) |  | 10(5.5%) | 7(10.3%) |  |

Abbreviations: EF, ejection fraction.
